# Comparing Groundwater Sampling Devices for Denitrification Assessment Using the N_2_ /Ar Method

**DOI:** 10.1111/gwat.70047

**Published:** 2026-01-29

**Authors:** Felix Fahrenbach, Thomas R. Rüde

**Affiliations:** ^1^ RWTH Aachen University Institute of Hydrogeology Lochnerstr. 4‐20 52064 Aachen Germany

## Abstract

The N_2_/Ar method is valuable for studying legacy nitrate and denitrification progress in groundwater systems. It uses dissolved N_2_ and Ar concentrations to calculate the amount of N_2_ originating from denitrification (excess‐N_2_). Successfully applying the method requires accurate values of N_2_ and Ar concentrations. Therefore, avoiding degassing and atmospheric contamination during groundwater sampling is crucial for reliable results. In this study, we focus on the effect of sampling devices on N_2_, Ar, and the resulting excess‐N_2_ concentrations. To evaluate this effect over a wide concentration range, we sampled 14 observation wells. One sample was collected using a submersible pump and another using a bladder pump. Furthermore, we collected multiple samples with both pumps at a fifteenth site to assess reproducibility. Additionally, we used a point‐source bailer for sampling at this site. The major ion concentrations show that the sampling device does not significantly influence the sample chemistry. In contrast, the measured N_2_, Ar, and calculated excess‐N_2_ concentrations significantly differ between the sampling devices. Overall, the samples collected with the submersible pump show the highest N_2_ and Ar concentrations, resulting in the highest excess‐N_2_ concentrations. N_2_ and Ar concentrations of the bladder pump samples are lower, resulting in lower excess‐N_2_ concentrations. The bailer samples show lower N_2_ but similar Ar concentrations to the submersible pump samples, leading to the lowest excess‐N_2_ concentrations. We conclude that a submersible pump is practical and suitable for collecting groundwater samples to assess denitrification by the N_2_/Ar method.

## Graphical abstract

Comparing N_2_, Ar, and excess‐N_2_ concentrations of groundwater samples collected with different devices shows that N_2_, Ar, and excess‐N_2_ concentrations significantly differ between the sampling devices. Using an electrical submersible pump for sampling yields the highest N_2_, Ar, and excess‐N_2_ concentrations.

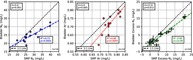

## Introduction

Microbial denitrification is a major pathway for removing nitrate (NO_3_^−^) from groundwater systems (Böhlke 2002). While suitable electron donors, either organic (chemo‐organotropic) or inorganic (chemo‐lithotrophic), oxidize during denitrification, nitrate reduces to elemental nitrogen (N_2_), which dissolves in groundwater (Korom 1992; Rivett et al. 2008). The N_2_/Ar method uses N_2_ and Ar concentrations to quantify the amount of N_2_ resulting from denitrification, called excess‐N_2_ (Weymann et al. 2008). Assuming nitrate, possible intermediate products, and other denitrification products (e.g., sulfate, bicarbonate) are also measured, this method allows identifying the denitrification type, assessing reaction progress, and quantifying initial nitrate concentrations (Tesoriero et al. 2000; Weymann et al. 2008; Jahangir et al. 2013; Zhou et al. 2018). Therefore, the N_2_/Ar method is a valuable tool widely applied when studying denitrification in groundwater systems (Blicher‐Mathiesen et al. 1998; Tesoriero et al. 2000; Mookherji et al. 2003; Green et al. 2008; Weymann et al. 2008; Kennedy et al. 2009; Jahangir et al. 2012, 2013; Young et al. 2013; McAleer et al. 2017; Zhou et al. 2018; Martindale et al. 2019; Hinshaw et al. 2020).

In past studies that applied the N_2_/Ar method, groundwater sampling was performed using different devices such as peristaltic pumps (Blicher‐Mathiesen et al. 1998; Weymann et al. 2008; Kennedy et al. 2009; Houben et al. 2018; Zhou et al. 2018; Hinshaw et al. 2020), submersible pumps (Weymann et al. 2008; Young et al. 2013; Houben et al. 2018; Martindale et al. 2019), bladder pumps (Jahangir et al. 2012, 2013), and bailers (Mookherji et al. 2003; McAleer et al. 2017). However, as far as we know, no study explicitly evaluates the effect of the devices used for groundwater lifting on the resulting N_2_ and Ar concentrations, which are crucial for accurate results from the N_2_/Ar method. To close this gap, we address the following questions with this study:

- Does the sampling device influence the overall water chemistry, especially parameters related to denitrification (e.g., nitrate, sulfate, bicarbonate)?
- Does the sampling device affect N_2_, Ar, and excess‐N_2_ concentrations?

Answering these questions and establishing standard sampling methods will benefit future studies that apply the N_2_/Ar method for denitrification assessment, which is crucial to maintaining groundwater quality.

## Materials and Methods

To span a wide range of dissolved N_2_, Ar, and excess‐N_2_, we sampled 14 observation wells. At each well, one sample was collected with an electrical centrifugal submersible pump (Grundfos MP1; frequency‐controlled) and one with a bladder pump (Solinst Model 407), operated using a pneumatic controller (Solinst Model 464, 250 psi). Repeatability was assessed at a fifteenth well by collecting multiple samples with both pumps and an additional point‐source bailer (Solinst Model 429).

The observation wells are located in the Lower Rhine Embayment (Western Germany), a Cenozoic multilayer aquifer system composed of unconsolidated marine and fluvial sediments (Schäfer and Utescher 2014). The wells are screened in suboxic to anoxic groundwater at depths of 38–120 m. Nitrate influx into deeper aquifers happens through hydraulic windows. The sampled wells are located at varying distances from possible influx areas. The deeper aquifers are known for their pyrite content, enabling chemo‐lithotrophic denitrification (Mäurer and Wisotzky 2008).

### Groundwater Sampling and Analysis

Before groundwater sampling, observation wells were purged with the electrical submersible pump until the stagnant water columns were replaced at least twice and on‐site parameters stabilized (specific electrical conductivity E.C., water temperature T, pH, dissolved oxygen D.O., and redox potential Eh), measured using WTW‐Xylem electrodes in a closed flow‐through cell. After purging, unfiltered samples were collected using the three sampling devices in the following order: (1) electrical submersible pump (Q ~ 2–3 L/min), (2) bladder pump (Q ~ 0.1–0.2 L/min), and (3) point‐source bailer, which was only used at observation well no. 15.

With each sampling device, we filled two types of sample bottles: (1) 500 mL high‐density polyethylene bottles and (2) triplicates of 100 mL glass crimp cap vials, filled following the USGS Groundwater Dating Laboratory guidelines (see Figure S1). All samples were kept cool (~6°C) until further laboratory analysis. The samples were transported by car to the laboratory (approx. 350 km). During transportation, the samples were kept cool using portable cooling boxes. In three vials (bladder pump samples), we observed the formation of a small (~100 μL) gas bubble during storage.

Major ions were analyzed to verify consistency in water type across sampling devices. Na^+^, K^+^, Ca^2+^, Mg^2+^, Cl^−^, NO_3_^−^, and SO_4_^2−^ were quantified by ion chromatography (Metrohm 790 Personal IC) on filtered (0.45 μm) aliquots of the 500 mL samples. Bicarbonate (HCO_3_^−^) was calculated from acid‐neutralizing capacity (Gran method) determined by digital titration (Hach Lange) on unfiltered aliquots; titration data were analyzed with the USGS Alkalinity Calculator (v2.22). Analytical precision and accuracy of analytical methods were ±5%; ion balances were within ±5% for all samples except one (−9%).

The dissolved gasses N_2_ and Ar were quantified on the 100 mL samples. Duplicate samples were analyzed, and their N_2_ and Ar concentrations were averaged. Dissolved N_2_ and Ar concentrations were measured using a modified membrane‐inlet mass spectrometer (Kana et al. 1994) at the GEO‐data Laboratory (Garbsen, Germany). The extended measurement uncertainties (*k* = 2) were 6.6% for N_2_ and 6.1% for Ar.

### Calculation of Excess‐N_2_

The following calculations are based on molar concentrations. According to Weymann et al. (2008), excess‐N_2_ can be calculated by subtracting atmospheric sources—nitrogen from equilibrium with soil air ($X_{N_{2},\mathrm{EQ}}$) and dissolution of entrapped soil air bubbles (excess‐air, $X_{N_{2},\mathrm{EA}}$)—from the total nitrogen amount in a groundwater sample ($X_{N_{2},\mathrm{obs}}$):

(1) $$X_{\text{excess}-N_{2}}=X_{N_{2},\mathrm{obs}}-X_{N_{2},\mathrm{EA}}-X_{N_{2},\mathrm{EQ}}$$

Due to fractionation during the dissolution of entrapped air bubbles, uncertainty is assigned to the excess‐air component when only one noble gas (e.g., Ar) is measured. To assess this uncertainty, two boundary scenarios can be calculated (Weymann et al. 2008). The first (Equation 2) assumes a complete dissolution of entrapped air bubbles; the second (Equation 3) describes the case when minimal—nearly zero—dissolution of the air bubbles occurs.

(2) $$X_{N_{2},\mathrm{EA}}=\left(X_{\mathrm{Ar},\mathrm{obs}}-X_{\mathrm{Ar},\mathrm{EQ}}\right)\cdot \frac{X_{N_{2},\mathrm{atm}}}{X_{\mathrm{Ar},\mathrm{atm}}}$$

(3) $$X_{N_{2},\mathrm{EA}}=\left(X_{\mathrm{Ar},\mathrm{obs}}-X_{\mathrm{Ar},\mathrm{EQ}}\right)\cdot \frac{X_{N_{2},\mathrm{EQ}}}{X_{\mathrm{Ar},\mathrm{EQ}}}$$

Equations 2 and 3 allow calculating an upper and lower estimate of excess‐N_2_. The mean of the upper and lower estimates is considered the best excess‐N_2_ estimate (Weymann et al. 2008).

According to Heaton and Vogel (1981), the mean annual air temperature is a reasonable estimate for groundwater recharge temperature. Therefore, we assumed a groundwater recharge temperature of 10°C, which results in equilibrium concentrations of 17.7 mg/L (0.6321 mmol/L) N_2_ and 0.67 mg/L (0.0168 mmol/L) Ar, calculated using the Bunsen coefficients provided by Weiss (1970). The molar N_2_/Ar ratio thus is 37.6. The atmospheric molar N_2_/Ar ratio is 83.6, based on volumetric fractions of 78.084% N_2_ and 0.934% Ar (Berner and Berner 2012).

The sum of excess‐N_2_, measured nitrate, and potential intermediate denitrification products yields the initial nitrate concentration at the point of groundwater recharge, ignoring denitrification in soil and vadose zone. The concentration ratio of denitrification products to initial nitrate provides an estimate of reaction progress (Weymann et al. 2008).

### Statistical Data Evaluation

We used standard metrics for descriptive data analysis, such as mean, median, standard deviation (sd), and coefficient of variation (CV). Data normality and variance homogeneity were checked using the Shapiro–Wilk test and Q‐Q plots, and Levene's and Fligner‐Killeen tests, respectively.

Differences in major ions, N_2_, Ar, and excess‐N_2_ concentrations among the sampling devices were evaluated using both parametric (paired t‐test, Welch's analysis of variance) and non‐parametric (paired Wilcox test, Kruskal‐Wallis test) methods. As multiple comparison tests (MCT), we calculated Tukey's Honest Significant Differences and Games‐Howell tests following the analysis of variance; pairwise Wilcox tests and Dunn's tests followed Kruskal‐Wallis tests. For the non‐parametric MCTs, p‐values were adjusted using the Benjamini‐Hochberg correction. Here, we focus on non‐parametric results, which are usually more robust (Helsel et al. 2020). Please see the Supporting Information for the results of the other tests.

All statistical calculations were performed using *R* (version: 4.4.2) and *Rstudio* (version: 2024.09.1 Build 394). The figures displaying the different data were created using *Python* (version: 3.11.4) scripts.

## Results

### Multiple Locations—Concentration Ranges

**Figure 1:**
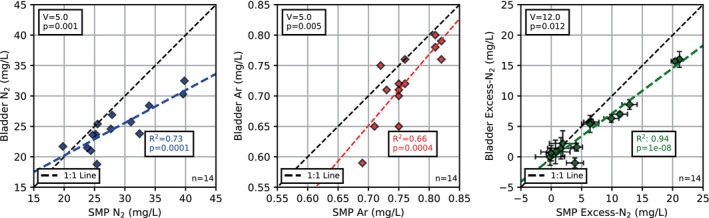
Comparison of dissolved N_2_, Ar, and excess‐N_2_ concentrations in samples collected at observation wells 1 to 14. The test statistics (V), the corresponding p‐values of paired Wilcox tests, the coefficients of determination (R^2^), and the p‐values of linear regressions are provided. The error bars indicate excess‐air uncertainty (based on Equations 2 and 3; expressed in mg/L). SMP, submersible pump.

Both pumping methods used at sites 1 to 14 yield similar major ion concentrations (paired Wilcox tests p‐values >0.05; see Figure S2). However, differences in N_2_, Ar, and excess‐N_2_ concentrations between the two pumping methods are evident (paired Wilcox tests p‐values <0.05; see Figure 1).

The samples collected with the submersible pump contain higher concentrations of all three gasses. For N_2_, the difference is more prominent for higher N_2_ concentrations, as indicated by the linear regression line (see Figure 1), which is rotated clockwise from the 1:1 line. For Ar, the difference is independent of Ar concentration, as indicated by a regression line that lies parallel to the 1:1 line. Excess‐N_2_ is calculated from the measured N_2_ and Ar concentrations and is the parameter of most interest. At low concentrations, both sampling devices yield similar excess‐N_2_ concentrations. At higher concentrations, the submersible pump leads to higher excess‐N_2_ concentrations, as indicated by a slightly clockwise rotation—compared to the 1:1 line—of the linear regression line in Figure 1.

### Single Location—Repeatability

**Figure 2:**
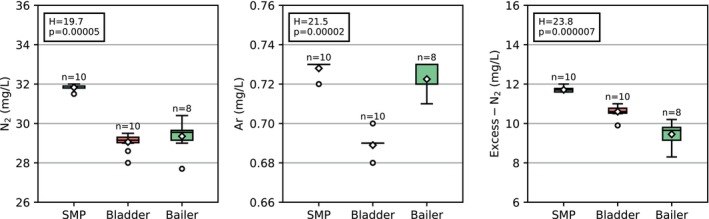
Comparison of dissolved N_2_, Ar, and excess‐N_2_ concentrations of samples collected at observation well 15. The boxes extend from the 25th to the 75th percentile; the whiskers extend to the farthest data point lying within 1.5 times the interquartile range from the box ends. All data points outside the whiskers are considered outliers (closed circles). The medians are the solid line within the boxes; the diamonds symbolize the means. The test statistics (H) and p‐values of the Kruskal‐Wallis tests are provided. SMP, submersible pump.

All three sampling devices that were used at site no. 15 yield the same general water type: nitrate‐free, with calcium as the dominant cation and sulfate as the dominant anion (see Table S1). Only the bailer sample shows lower sulfate and chloride concentrations, while the bicarbonate concentration is higher than that of the submersible and bladder pump samples. However, significant differences in N_2_, Ar, and excess‐N_2_ exist among the three sampling devices (Kruskal‐Wallis tests p‐values <0.05; see Figure 2).

The submersible pump produces the highest N_2_ concentrations that differ from those obtained by the bladder pump and the bailer (Dunn's MCT p‐values <0.05). The bladder pump and the bailer result in similar N_2_ concentrations (Dunn's MCT p‐value = 0.32). The spread of the submersible pump samples is the smallest (sd = 0.14 mg/L, CV = 0.4%) compared to the spread of the bladder pump (sd = 0.46 mg/L, CV = 1.6%) and bailer samples (sd = 0.79 mg/L, CV = 2.7%).

The submersible pump and the bailer yield similar Ar concentrations (Dunn's MCT p‐value = 0.28). While the spread of the submersible pump samples is minimal (sd = 0.004 mg/L, CV = 0.6%), the bailer samples show a more considerable variation (sd = 0.01, CV = 1.0%). The Ar concentrations of the bladder pump samples also show a minimal spread, disturbed by two outliers (sd = 0.01, CV = 0.8%). The bladder pump produces smaller Ar concentrations than the submersible pump and the bailer (Dunn's MCT p‐values <0.05).

All three sampling devices lead to different excess‐N_2_ concentrations (Dunn's MCT p‐values <0.05). The submersible pump samples show the highest excess‐N_2_ concentrations, approximately 1 mg/L higher than the bladder pump samples and 2 mg/L higher than the bailer samples. Excess‐N_2_ concentrations of the bladder pump samples are approximately 1 mg/L higher than those of the bailer samples, which show the lowest concentration with the highest spread (sd = 0.60 mg/L, CV = 6.3%). The submersible and bladder pump samples show low spreads (sd = 0.13 mg/L, CV = 1.1% and sd = 0.29 mg/L, CV = 2.7%).

## Discussion

### Study Limitations

Because we sampled observation wells in a real‐world aquifer system, the “real” concentrations of N_2_, Ar, and excess‐N_2_ are unknown. Consequently, even the highest apparent recovery of N_2_ and Ar might underestimate the “real” concentrations due to degassing during pumping, vial filling, and sample storage. This limitation might be addressed in future studies, e.g., by employing an (artificial) groundwater body with known N_2_ and Ar concentrations or by including more noble gasses, as noble gas ratios might reveal degassing processes. We assume it is improbable that one of the sampling devices introduced additional N_2_ or Ar into the samples. Therefore, we consider the highest N_2_ and Ar concentrations to be the ones closest to the “real” concentrations, as gas losses are the primary concern when sampling for dissolved N_2_ and Ar.

The present study focuses on the impact of sampling devices on dissolved N_2_ and Ar concentrations, using standard vials and storage procedures; an assessment of different sample containers and storage effects is beyond the scope of this study. The use of pressure‐proofed storage devices, such as clamped copper tubes, might be beneficial for dissolved gas analysis because such tubes allow maintaining positive pressure during sample filling by restricting the outflow at the tube end (Massmann et al. 2009; Sültenfuß et al. 2009). However, the use of non‐standard, specialized sample vials needs to be done in close cooperation with the analyzing laboratory.

Effects of sample containers, storage, and transportation can be eliminated when performing on‐site, online gas analysis using a portable inlet mass spectrometer (Brennwald et al. 2016). However, degassing effects attributed to the device used for groundwater lifting would also affect on‐site measurements. Because we focus on gas losses attributed to the used sampling devices, comparing the results of on‐site gas analysis with those from laboratory analysis is out of the study's scope.

As described above, the excess‐N_2_ concentrations are uncertain. We addressed this problem by calculating an upper and lower estimate of excess‐N_2_. This procedure is widely applied when only one noble gas (e.g., Ar) is measured (Weymann et al. 2008; Jahangir et al. 2012, 2013; Young et al. 2013; McAleer et al. 2017). The resulting uncertainty only slightly affects the overall implications of this study (e.g., see the error bars in Figure 1).

### Operational Pros and Cons of each Sampling Technique

An electrical submersible pump can be used for well purging and sampling, especially if the motor frequency is controllable. In case of the N_2_/Ar method, a high pumping rate can be used for fast well purging and a low pumping rate for sampling. Therefore, this method can be very time‐efficient. On the other hand, if the groundwater level is very deep, electrical submersible pumps might only achieve a low flow rate or be unable to lift the groundwater. For example, the centrifugal submersible pump we used in this study (Grundfos MP1) can lift water from depths up to 90 m, but in that case, the observed maximum flow rate is approximately 6 to 7 L/min. Operating an electrical submersible pump requires a power supply, which may not always be available.

Bladder pumps allow sampling from deeper observation wells; for example, the pump used in this study (Solinst Model 407) can sample to depths of up to 150 m below ground surface. A bladder pump's low flow rate may result in long or impractical purging times. If a compressor is used for compressed gas delivery, the operation of a bladder pump also requires a power supply; if prefilled gas tanks are used, a bladder pump can be used without an external power supply.

Point‐source bailers can be used for very deep sampling, as they are limited only by available rope length. We used a stainless steel point‐source bailer (Solinst Model 429). Another advantage of bailers is that they are available with small diameters, allowing for sampling observation wells with diameters smaller than DN 50 or 2 in. As no additional equipment is needed, using bailers can be advantageous for sample collection in terrains with limited accessibility and no electrical power supply. In general, well purging is not possible using a bailer. The bailer's volume and discontinuous water delivery might impede sample collection, especially since the procedure for crimp cap vial filling (see Figure S1) is challenging to accomplish if the bailer's volume is insufficient.

### Differences in Dissolved Gas Concentrations

By determining dissolved N_2_ and Ar concentrations in groundwater samples, the N_2_/Ar method aims to quantify the amount of N_2_ resulting from denitrification (excess‐N_2_), which is used to calculate initial nitrate concentration and assess reaction progress (Weymann et al. 2008). The results show that measured N_2_, Ar, and the resulting excess‐N_2_ concentrations differ between the sampling techniques. Because the sampling devices yield the same general water type at each observation well after purging using the submersible pump, indicated by similar major ion concentrations, these differences cannot be attributed to differences in the sampled groundwater. Consequently, we infer that the different sampling devices are, to varying degrees, susceptible to sample degassing.

The electrical centrifugal submersible pump appears less prone to gas losses because it yields the highest concentrations of N_2_, Ar, and excess N_2_. The pump's continuous water flow allows quick vial filling, minimizing air contact time. Because the pump applies positive pressure, the pumped water remains under sufficient pressure, reducing the risk of sample degassing from pressure losses. The resulting dissolved gas concentrations are highly reproducible (see Figure 2). Earlier studies from the 1980s, reviewed by Parker (1994), document a significant loss of volatile compounds when using submersible pumps. However, the submersible pump's flow rates were not adjustable at that time. Studies including a submersible pump that allows adjustment of the flow rate have also reported good recovery rates of volatile compounds (Knobel and Mann 1993; Gibs et al. 1994; Rivard et al. 2018).

Generally, bladder pumps are considered one of the best devices for groundwater sampling, especially when volatile compounds are of interest (Parker 1994). Several studies have found excellent recovery rates of volatile organic compounds using bladder pumps (Barcelona et al. 1984; Imbrigiotta et al. 1988; Tai et al. 1991; Rivard et al. 2018). However, comparing the results from submersible and bladder pump samples indicates that some losses of dissolved N_2_ and Ar can occur when using a bladder pump. Although we observed bubble formation in three vials from bladder‐pump samples, this degassing is unlikely to be attributed to the vials or storage procedures, as both were identical for all samples and no bubble formation was observed in the submersible‐pump and bailer samples. Because bladder pumps are designed for sampling volatiles, we think degassing occurs during vial filling, not during pumping. Due to the operating principle of bladder pumps—pressurizing the bladder during drive times and allowing it to relax during vent times—the discharge at the outlet of the sample line is discontinuous. During vent times, the delivered sample water experiences no positive pressure and is susceptible to degassing. This effect might be enhanced because filling and sealing the 100 mL vials according to the USGS recommended procedure (see Figure S1) requires multiple drive and vent cycles, providing more time for degassing. Very small, initially non‐visible gas bubbles introduced into the sample during vial filling may coalesce into a larger, observable bubble during storage. Adjusting the vial volume to the bladder pump's flow rate might reduce degassing during sample collection. On the other hand, smaller vials are more susceptible to atmospheric contamination during the laboratory analysis (Inglett et al. 2013). Therefore, the vial volume should also be selected in consultation with the laboratory.

While some studies have found good recovery rates of volatiles using a point‐source bailer (Barcelona et al. 1984; Muska et al. 1986; Imbrigiotta et al. 1988), others have observed significantly lower concentrations compared to control samples or samples obtained with another sampling device (Tai et al. 1991; Gibs et al. 1994). Because of its limited volume, following the sampling procedure for dissolved N_2_ and Ar (see Figure S1) with a bailer is challenging, potentially leading to gas losses, especially of the more volatile N_2_. We also noted that the bailer's outflow velocity varies depending on how deeply the user inserts the outlet device. This is reflected in the poorer reproducibility compared to the two pumping methods (see Figure 2). A higher variability of bailer samples was also observed in studies considering volatile organic compounds (Barcelona et al. 1984; Muska et al. 1986; Imbrigiotta et al. 1988; Tai et al. 1991).

The observed differences have profound implications when applying the N_2_/Ar method to study denitrification. If excess‐N_2_ is present (>2 mg/L), excess‐N_2_ concentrations from samples retrieved with the submersible pump are 1 to 5 mg/L higher than those collected with the bladder pump and approximately 2 mg/L higher compared to the point source bailer samples. A difference of 2 mg/L excess‐N_2_ would lead to an initial nitrate concentration difference of approximately 9 mg/L; a difference of 5 mg/L excess‐N_2_ would result in a difference of approximately 22 mg/L, almost half the well‐established groundwater guideline value of 50 mg/L nitrate. Therefore, the observed differences can have a substantial impact when assessing nitrate influxes and denitrification progress.

## Conclusion and Recommendations

Groundwater sampling for the analysis of dissolved N_2_ and Ar involves several stages. In this study, we focus on effects associated with different devices used to accomplish the first stage of sampling: lifting a certain volume of groundwater to the surface. We therefore compared three different devices for groundwater lifting while using identical standard sample vials and uniform storage and transport procedures for all samples. Degassing associated with the lifting device can affect both on‐site and off‐site analyses. Potential effects of storage and transport on N_2_ and Ar concentrations were beyond the study's scope but may be addressed in future work. Our results suggest that a submersible pump is a highly suitable device for N_2_ and Ar groundwater sampling. It can be used for well purging and sampling, especially if the motor frequency is controllable. Therefore, it is the most time‐efficient sampling technique and showed the highest apparent recovery of N_2_ and Ar; potential unknown underestimations should be evaluated in future studies. A bladder pump can be employed if sampling is necessary from depths too deep for a submersible pump, but well purging with a bladder pump might not be feasible. Due to the bladder pump's low flow rate, filling the sample vials takes longer than with the submersible pump, giving more time for gas losses and atmospheric contaminations. To avoid this effect, it might be beneficial to adjust the sample volume to the bladder pump's achievable flow rate. A bailer can retrieve samples from even larger depths, but no well purging is possible. The bailer volume might also limit sampling procedures, like allowing the sample vials to overflow several times and sealing the vials underwater to prevent gas loss and atmospheric contamination. Based on our data analysis, we recommend using a submersible pump for studies applying the N_2_/Ar method to assess denitrification.

## Authors' Note

The authors declare that there are no conflicts of interest relevant to this study.

## Supporting information


**Figure S1.** Filling of crimp cap vials for N_2_ and Ar analysis following the USGS Groundwater Dating Laboratory guidelines.
**Figures S2–S3.** Comparison of major ion concentrations in samples collected at observation wells 1 to 14.
**Figures S4–S14.** Q‐Q plot of major ion, N_2_, Ar, and excess‐N_2_ concentrations for observation wells 1 to 14.
**Figures S15–S25.** Q‐Q plots of major ion, N_2_, Ar, and excess‐N_2_ concentration differences (SMP‐Bladdder) for observation wells 1 to 14.
**Figures S26–S28.** Q‐Q pots of N_2_, Ar, excess‐N_2_ concentrations for observation well 15.
**Table S1.** Major Ion Concentrations of the Samples collected at Observation Well 15.
**Tables S2–S6.** Statistical Results regarding the comparison of major ion, N_2_, Ar, and excess‐N_2_ concentration for observation wells 1 to 14.
**Tables S7–S16.** Statistical Results regarding the comparison of major ion, N_2_, Ar, and excess‐N_2_ concentration for observation well 15.

## Data Availability

All the data, *R*, and *Python* scripts are available on HydroShare (Fahrenbach and Rüde 2026).
