# Supplementary material for: Comparing Groundwater Sampling Devices for Denitrification Assessment Using the N2 /Ar Method
Source: Ground Water. 2026 Jan 29;64(2):235–42. doi: 10.1111/gwat.70047 (PMC12990959; doi:10.1111/gwat.70047)
Supplement: Supplementary file 1 — Figure S1. Filling of crimp cap vials for N2 and Ar analysis following the USGS Groundwater Dating Laboratory guidelines. Figures S2–S3. Comparison of major ion concentrations in samples collected at observation wells 1 to 14. Figures S4–S14. Q‐Q plot of major ion, N2, Ar, and excess‐N2 concentrations for observation wells 1 to 14. Figures S15–S25. Q‐Q plots of major ion, N2, Ar, and excess‐N2 concentration differences (SMP‐Bladdder) for observation wells 1 to 14. Figures S26–S28. Q‐Q pots of N2, Ar, excess‐N2 concentrations for observation well 15. Table S1. Major Ion Concentrations of the Samples collected at Observation Well 15. Tables S2–S6. Statistical Results regarding the comparison of major ion, N2, Ar, and excess‐N2 concentration for observation wells 1 to 14. Tables S7–S16. Statistical Results regarding the comparison of major ion, N2, Ar, and excess‐N2 concentration for observation well 15. [file GWAT-64-235-s001.pdf]

Supporting Information for *Groundwater*

Comparing Groundwater Sampling Devices for Denitrification Assessment using the N_2/Ar Method

Felix Fahrenbach[1], Thomas R. Rüde[1]

[1]RWTH Aachen University, Institute of Hydrogeology, Aachen, Germany

Corresponding author: Felix Fahrenbach (fahrenbach@hydro.rwth-aachen.de)

Contents of this file

Figure S1. Filling of crimp cap vials for N_2 and Ar analysis following the USGS Groundwater Dating Laboratory guidelines.

Figures S2 to S3. Comparison of major ion concentrations in samples collected at observation wells 1 to 14.

Figures S4 to S14. Q-Q plot of major ion, N_2, Ar, and excess-N_2 concentrations for observation wells 1 to 14.

Figures S15 to S25. Q-Q plots of major ion, N_2, Ar, and excess-N_2 concentration differences (SMP-Bladdder) for observation wells 1 to 14.

Figures S26 to S28. Q-Q pots of N_2, Ar, excess-N_2 concentrations for observation well 15.

Table S1. Major Ion Concentrations of the Samples collected at Observation Well 15.

Tables S2 to S6. Statistical Results regarding the comparison of major ion, N_2, Ar, and excess-N_2 concentration for observation wells 1 to 14.

Tables S7 to S16. Statistical Results regarding the comparison of major ion, N_2, Ar, and excess-N_2 concentration for observation well 15.

Abbreviations

SMP – Submersible Pump

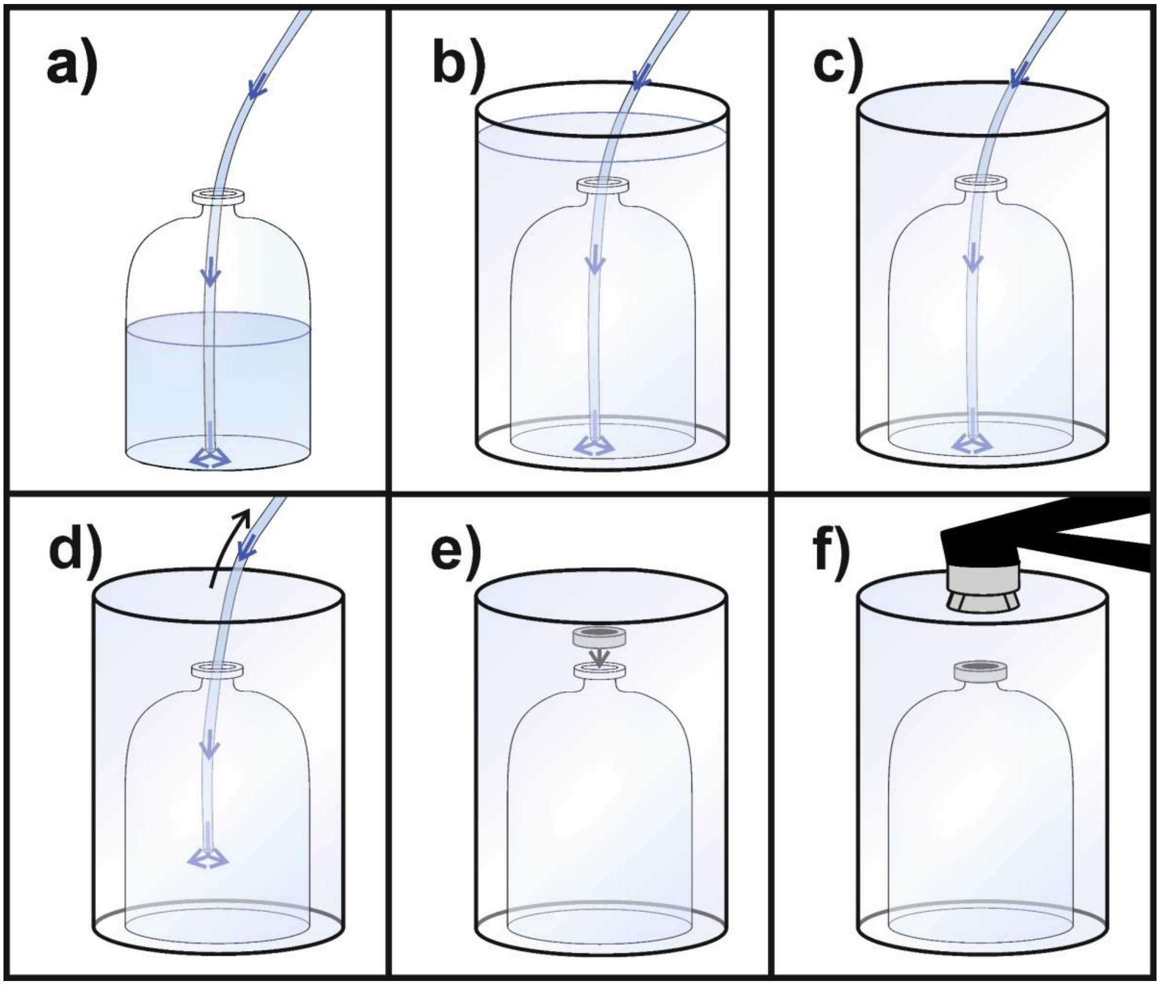

Figure S1. Filling of crimp cap vials for N_2 and Ar analysis following the USGS Groundwater Dating Laboratory guidelines (https://water.usgs.gov/lab/dissolved-gas/sampling/). a) Filling of a crimp cap vial. b) Placing the filled vial in a beaker filled with fresh groundwater. c) Allowing for 3x volume exchange. d) Removing the sample line slowly. e) Placing a crimp cap on top of the vial. f) Sealing the vial.

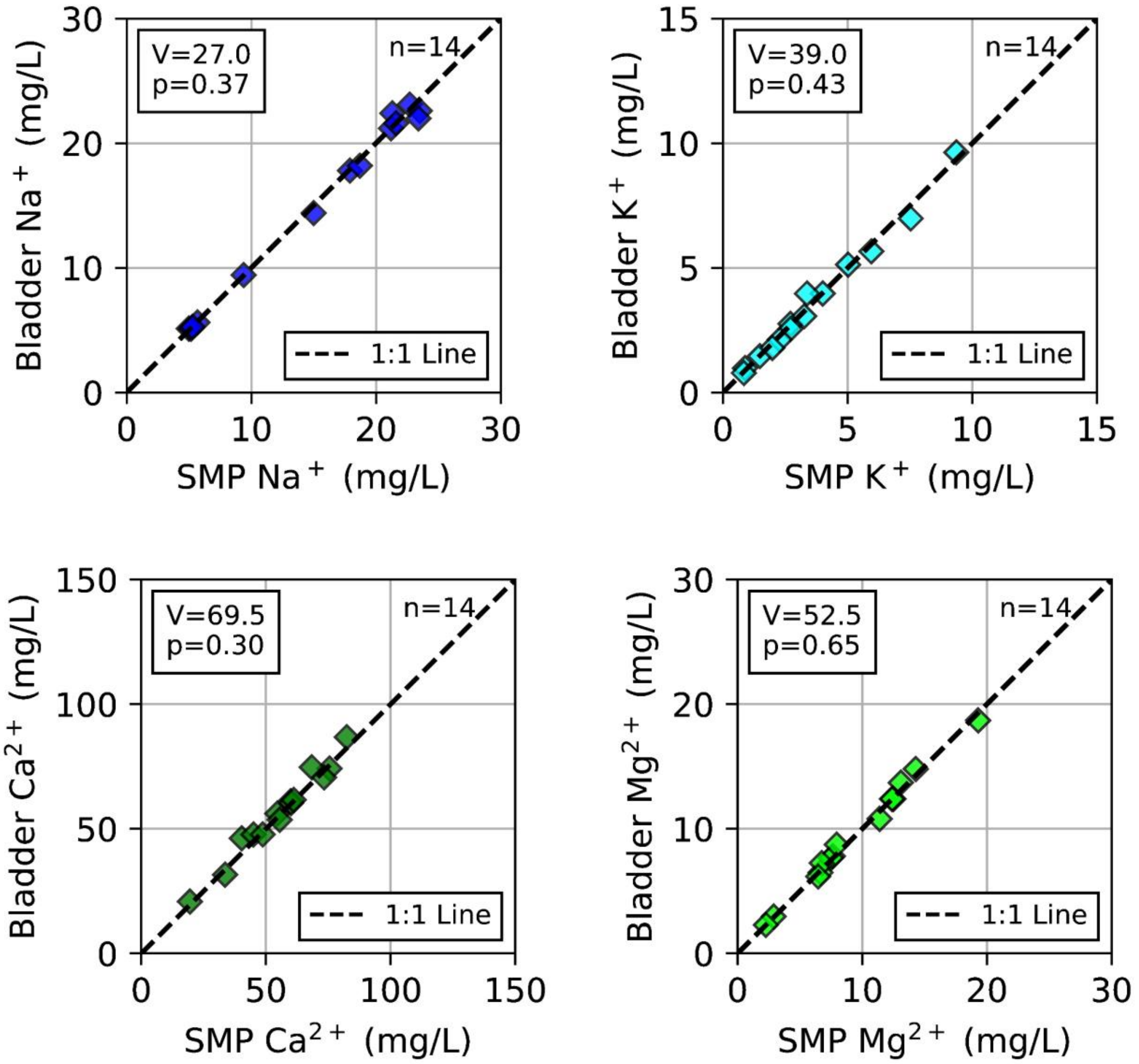

Figure S2. Comparison of major cations concentrations in samples collected at observation wells 1 to 14. The test statistics (V) and the corresponding p-values of paired Wilcox tests are provided. SMP – submersible pump.

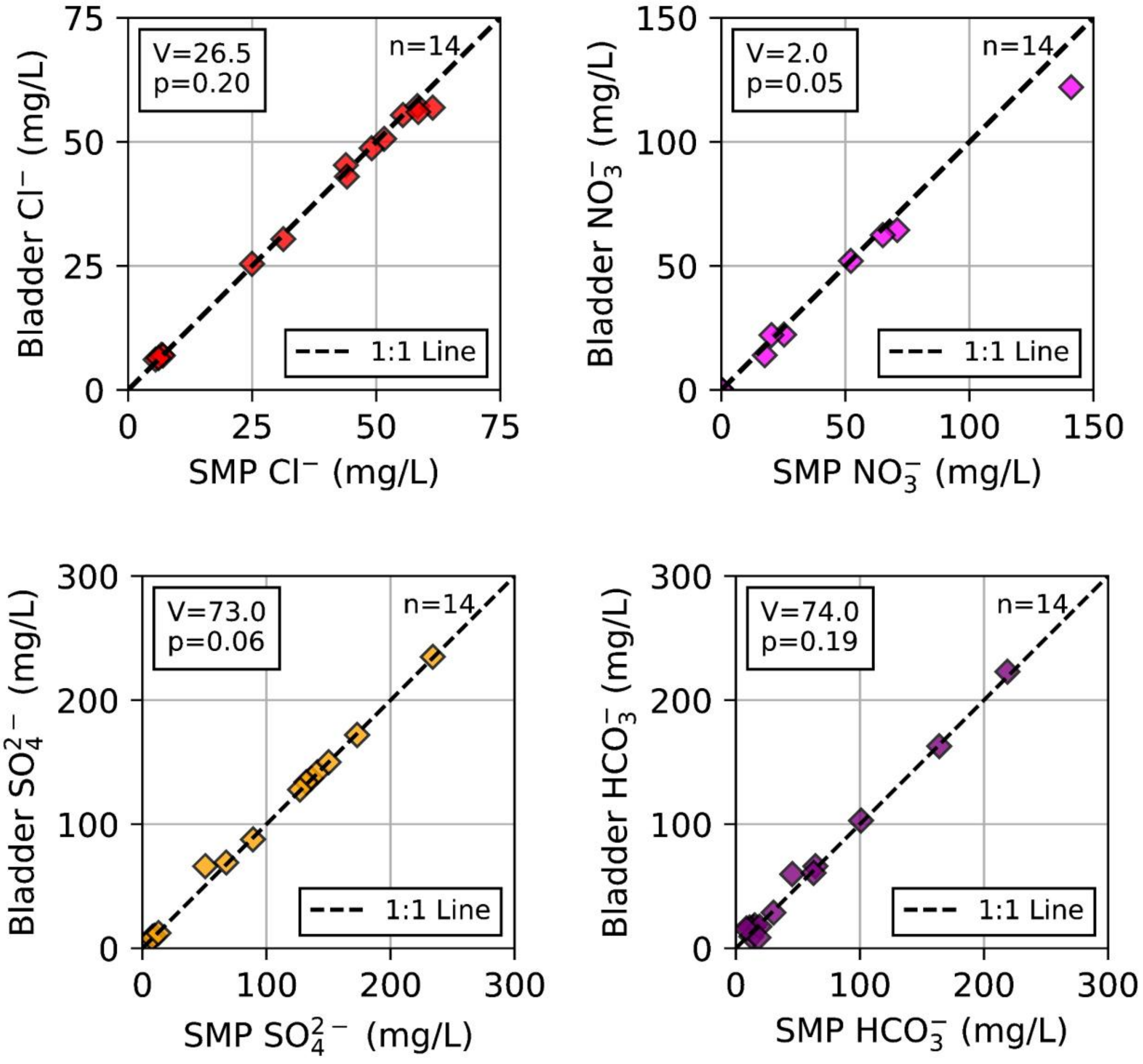

Figure S3. Comparison of major anions concentrations in samples collected at observation wells 1 to 14. The test statistics (V) and the corresponding p-values of paired Wilcox tests are provided. SMP – submersible pump.

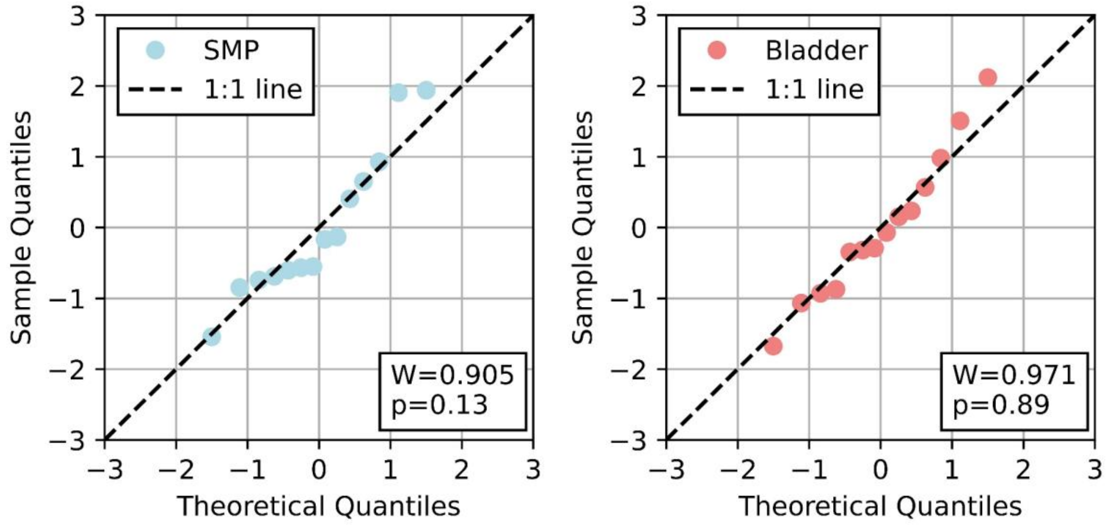

Figure S4. Q-Q plot of N_2 concentrations for observation wells 1 to 14. The test statistics (W) and the corresponding p-values of Shapiro-Wilk tests are provided.

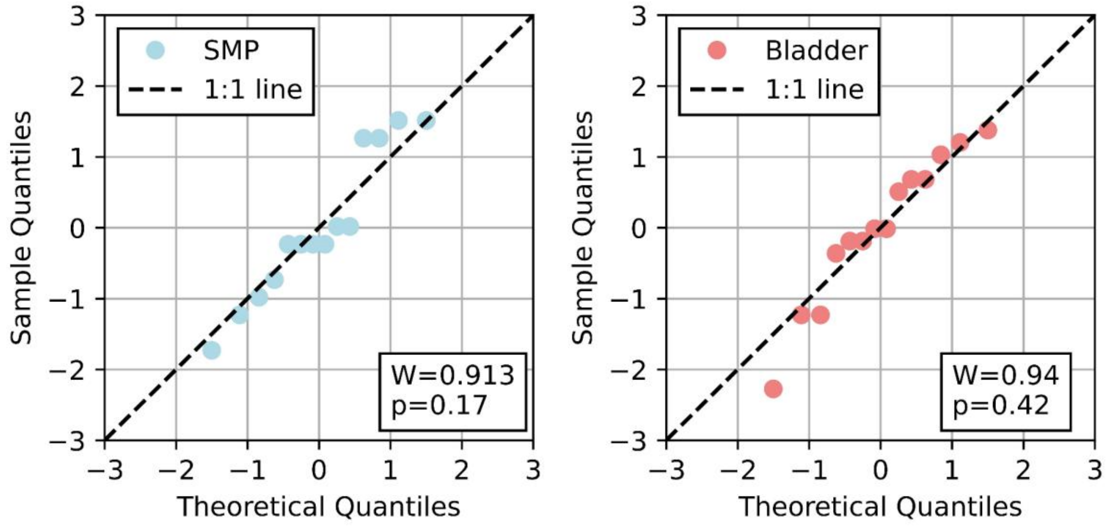

Figure S5. Q-Q plot of Ar concentrations for observation wells 1 to 14. The test statistics (W) and the corresponding p-values of Shapiro-Wilk tests are provided.

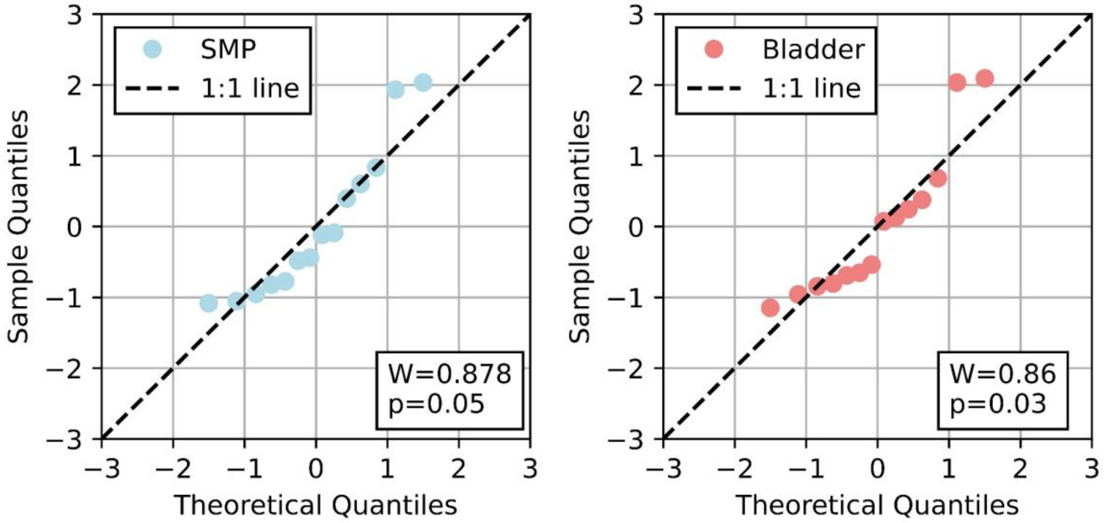

Figure S6. Q-Q plot of excess-N_2 concentrations for observation wells 1 to 14. The test statistics (W) and the corresponding p-values of Shapiro-Wilk tests are provided.

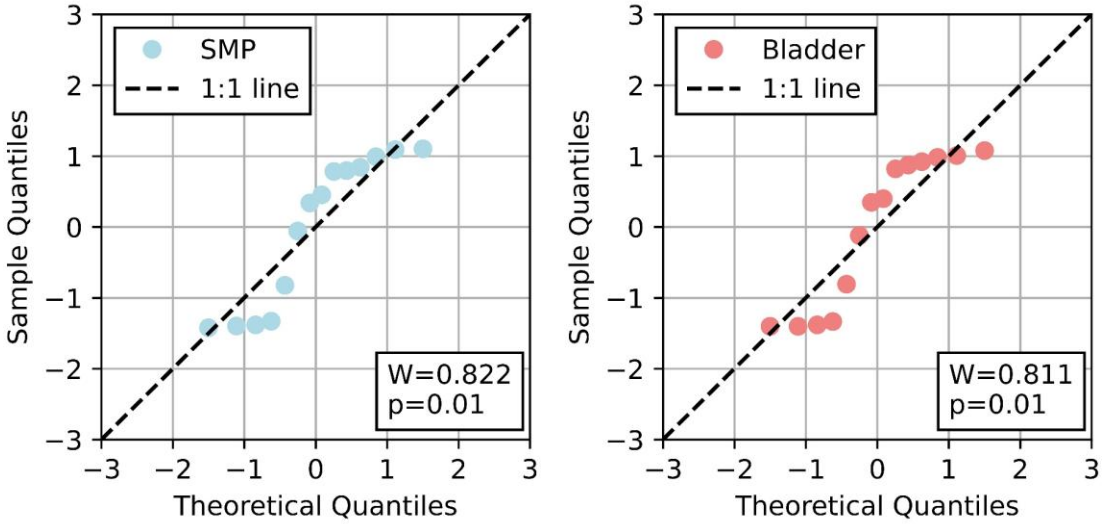

Figure S7. Q-Q plot of Na^+ concentrations for observation wells 1 to 14. The test statistics (W) and the corresponding p-values of Shapiro-Wilk tests are provided.

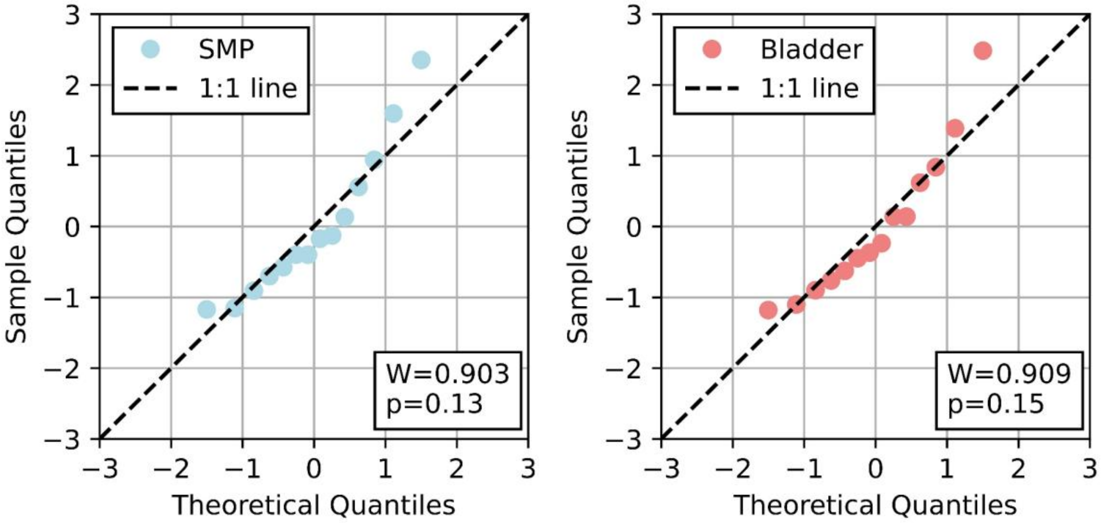

Figure S8. Q-Q plot of K^+ concentrations for observation wells 1 to 14. The test statistics (W) and the corresponding p-values of Shapiro-Wilk tests are provided.

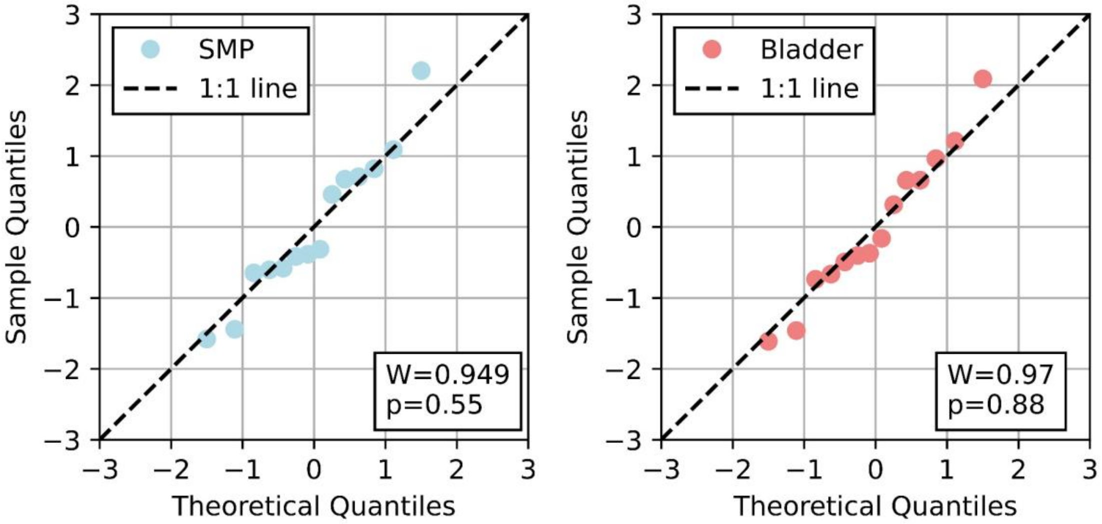

Figure S9. Q-Q plot of Mg^{2+} concentrations for observation wells 1 to 14. The test statistics (W) and the corresponding p-values of Shapiro-Wilk tests are provided.

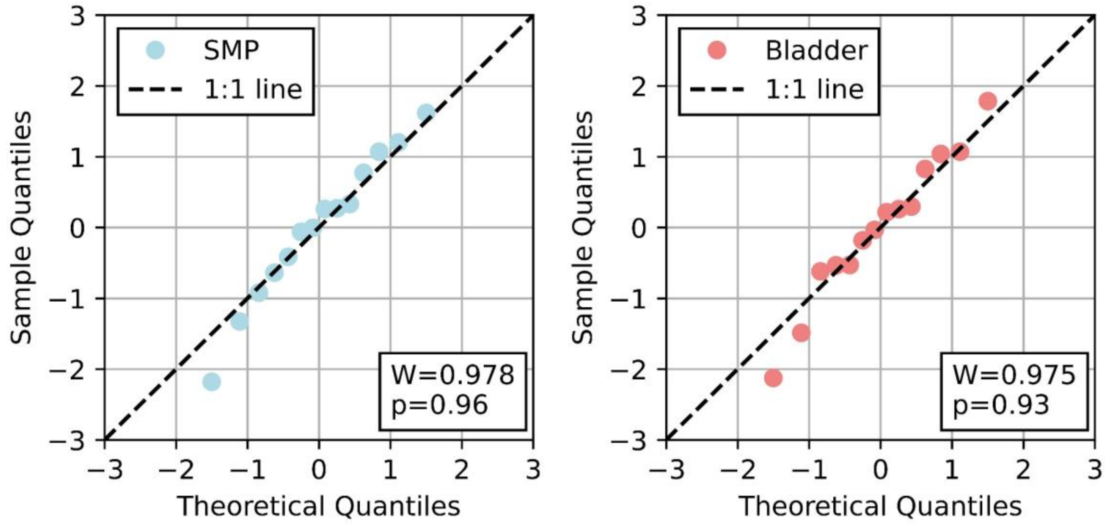

Figure S10. Q-Q plot of Ca^{2+} concentrations for observation wells 1 to 14. The test statistics (W) and the corresponding p-values of Shapiro-Wilk tests are provided.

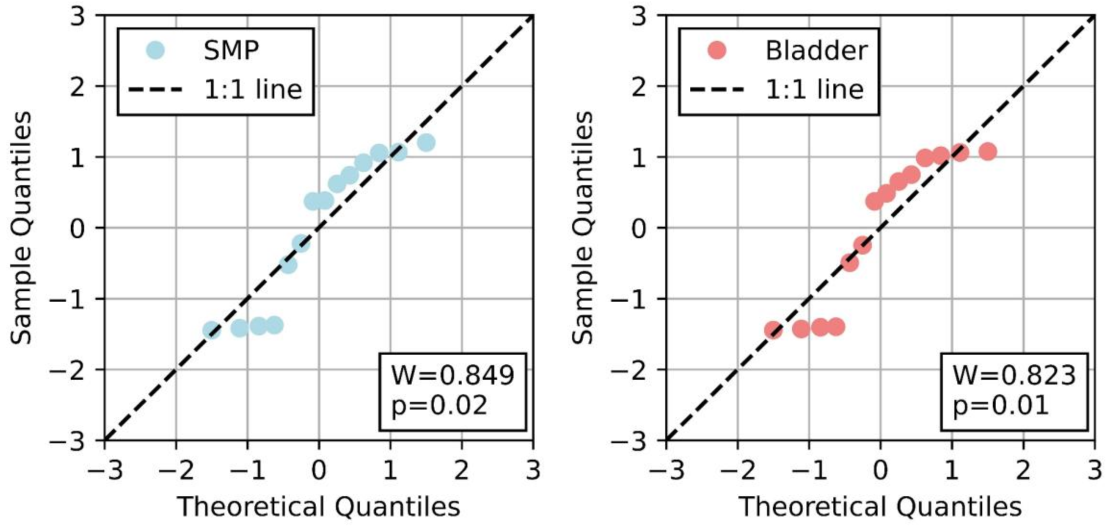

Figure S11. Q-Q plot of Cl^{-} concentrations for observation wells 1 to 14. The test statistics (W) and the corresponding p-values of Shapiro-Wilk tests are provided.

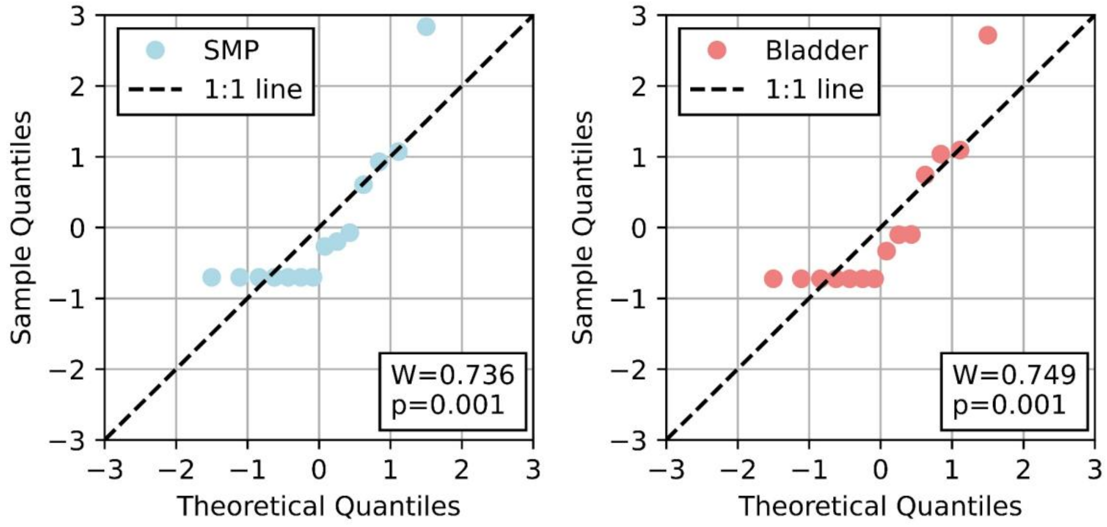

Figure S12. Q-Q plot of NO_3^- concentrations for observation wells 1 to 14. The test statistics (W) and the corresponding p-values of Shapiro-Wilk tests are provided.

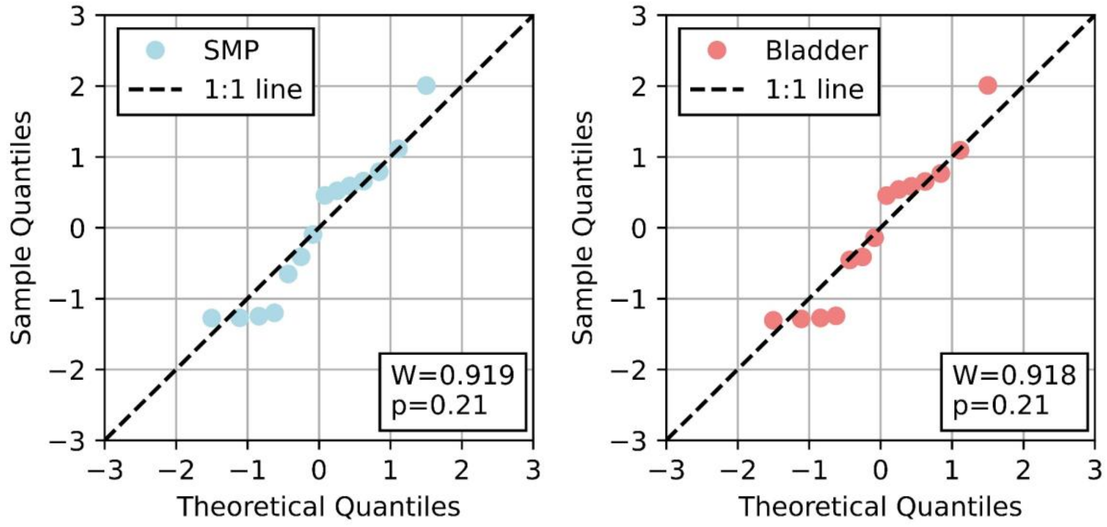

Figure S13. Q-Q plot of SO_4^{2-} concentrations for observation wells 1 to 14. The test statistics (W) and the corresponding p-values of Shapiro-Wilk tests are provided.

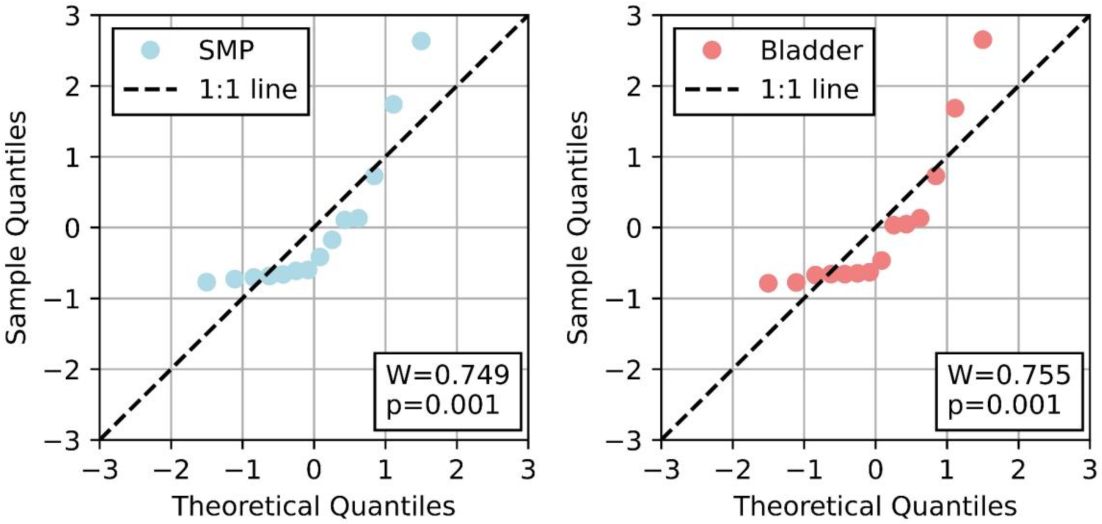

Figure S14. Q-Q plot of HCO_3^- concentrations for observation wells 1 to 14. The test statistics (W) and the corresponding p-values of Shapiro-Wilk tests are provided.

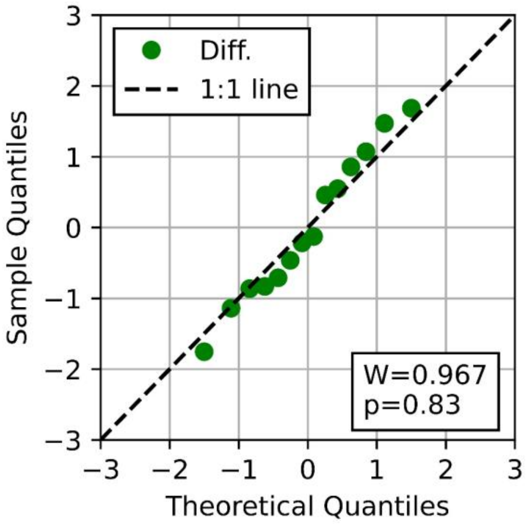

Figure S15. Q-Q plot of N_2 concentration differences (SMP-Bladder samples) for observation wells 1 to 14. The test statistics (W) and the corresponding p-values of Shapiro-Wilk tests are provided.

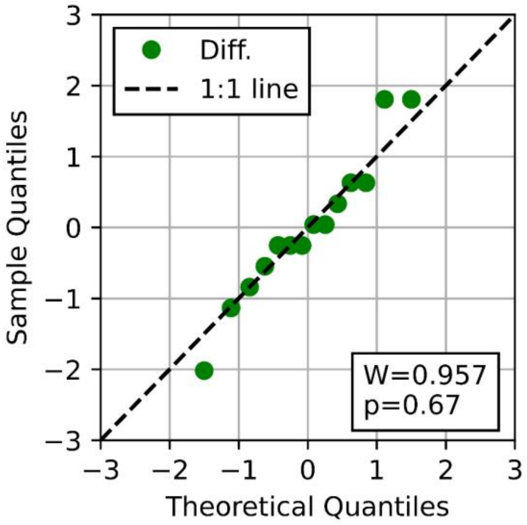

Figure S16. Q-Q plot of Ar concentration differences (SMP-Bladder samples) for observation wells 1 to 14. The test statistics (W) and the corresponding p-values of Shapiro-Wilk tests are provided.

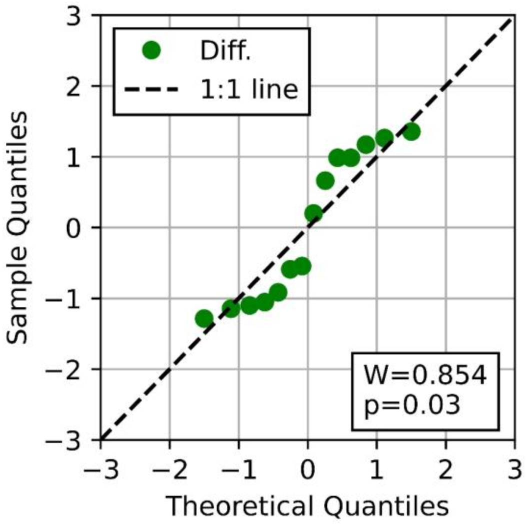

Figure S17. Q-Q plot of excess-N_2 concentration differences (SMP-Bladder samples) for observation wells 1 to 14. The test statistics (W) and the corresponding p-values of Shapiro-Wilk tests are provided.

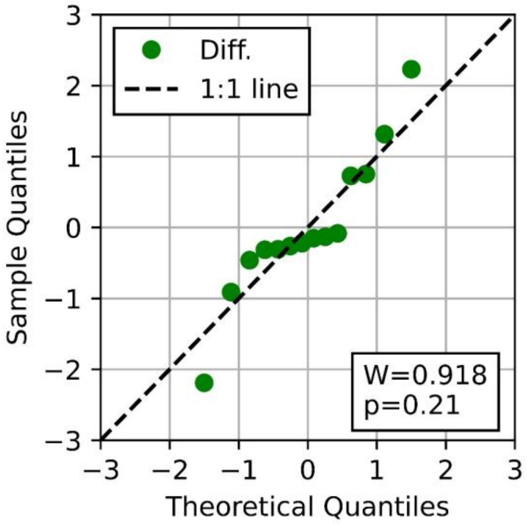

Figure S18. Q-Q plot of Na^+ concentration differences (SMP-Bladder samples) for observation wells 1 to 14. The test statistics (W) and the corresponding p-values of Shapiro-Wilk tests are provided.

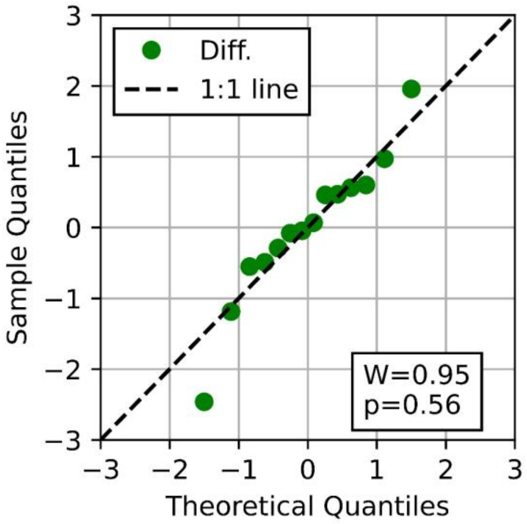

Figure S19. Q-Q plot of K^+ concentration differences (SMP-Bladder samples) for observation wells 1 to 14. The test statistics (W) and the corresponding p-values of Shapiro-Wilk tests are provided.

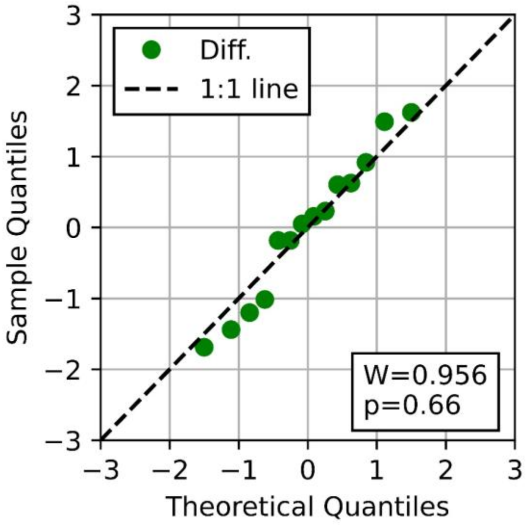

Figure S20. Q-Q plot of Mg^{2+} concentration differences (SMP-Bladder samples) for observation wells 1 to 14. The test statistics (W) and the corresponding p-values of Shapiro-Wilk tests are provided.

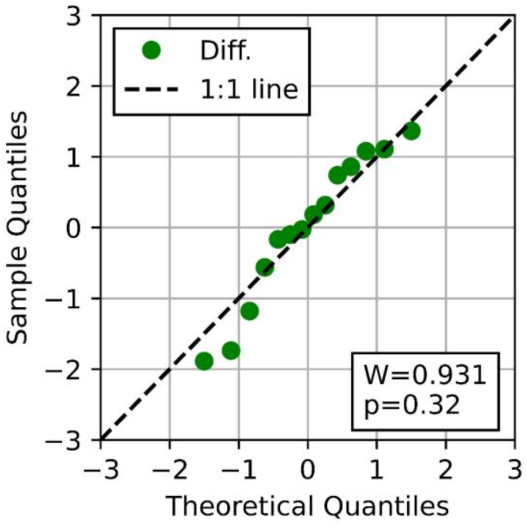

Figure S21. Q-Q plot of Ca^{2+} concentration differences (SMP-Bladder samples) for observation wells 1 to 14. The test statistics (W) and the corresponding p-values of Shapiro-Wilk tests are provided.

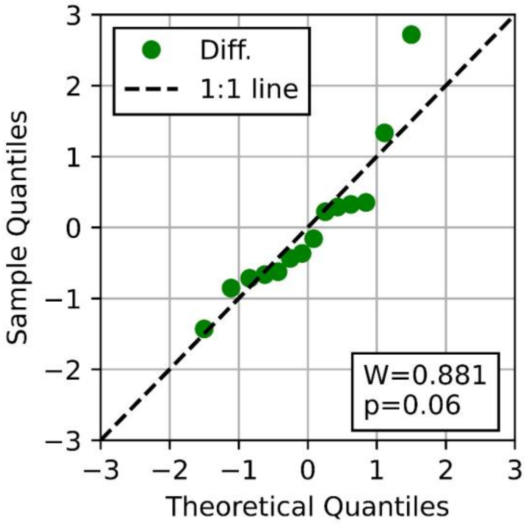

Figure S22. Q-Q plot of Cl^{-} concentration differences (SMP-Bladder samples) for observation wells 1 to 14. The test statistics (W) and the corresponding p-values of Shapiro-Wilk tests are provided.

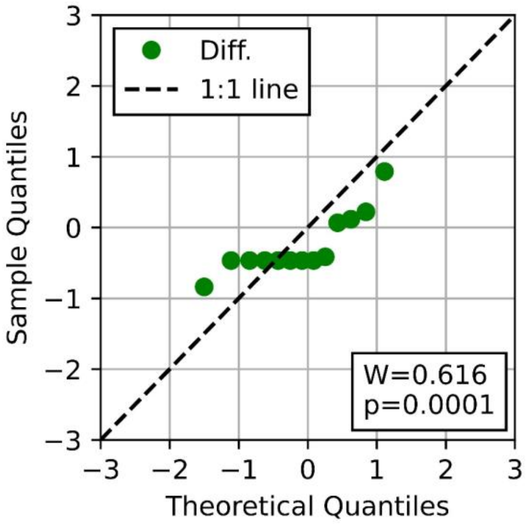

Figure S23. Q-Q plot of NO_3^- concentration differences (SMP-Bladder samples) for observation wells 1 to 14. The test statistics (W) and the corresponding p-values of Shapiro-Wilk tests are provided.

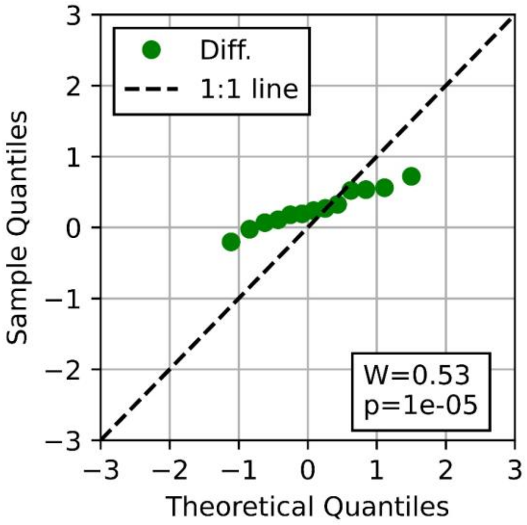

Figure S24. Q-Q plot of SO_4^{2-} concentration differences (SMP-Bladder samples) for observation wells 1 to 14. The test statistics (W) and the corresponding p-values of Shapiro-Wilk tests are provided.

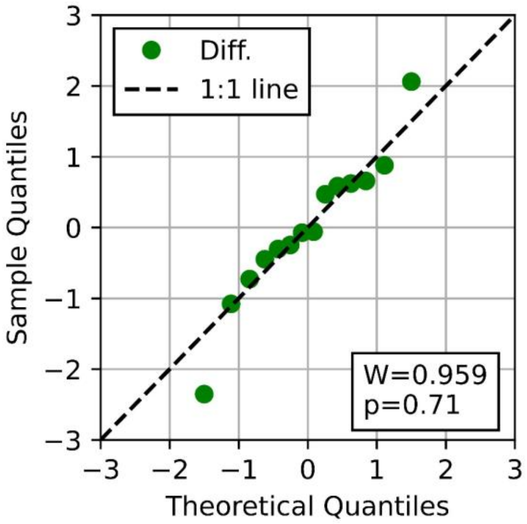

Figure S25. Q-Q plot of HCO_3^- concentration differences (SMP-Bladder samples) for observation wells 1 to 14. The test statistics (W) and the corresponding p-values of Shapiro-Wilk tests are provided.

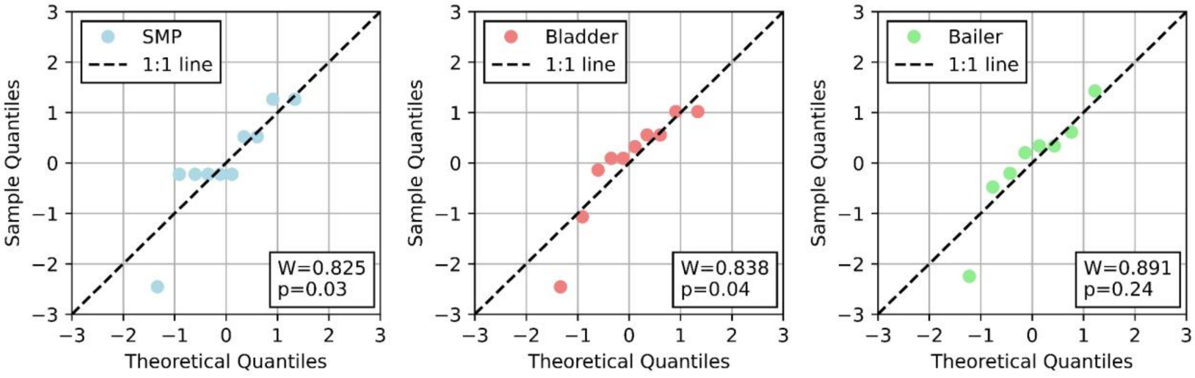

Figure S26. Q-Q plot of N_2 concentrations for observation well 15. The test statistics (W) and the corresponding p-values of Shapiro-Wilk tests are provided.

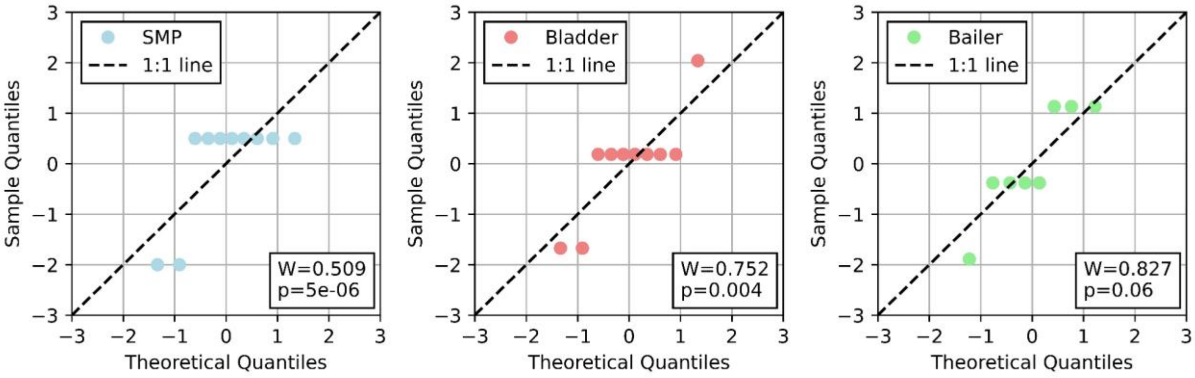

Figure S27. Q-Q plot of Ar concentrations for observation well 15. The test statistics (W) and the corresponding p-values of Shapiro-Wilk tests are provided.

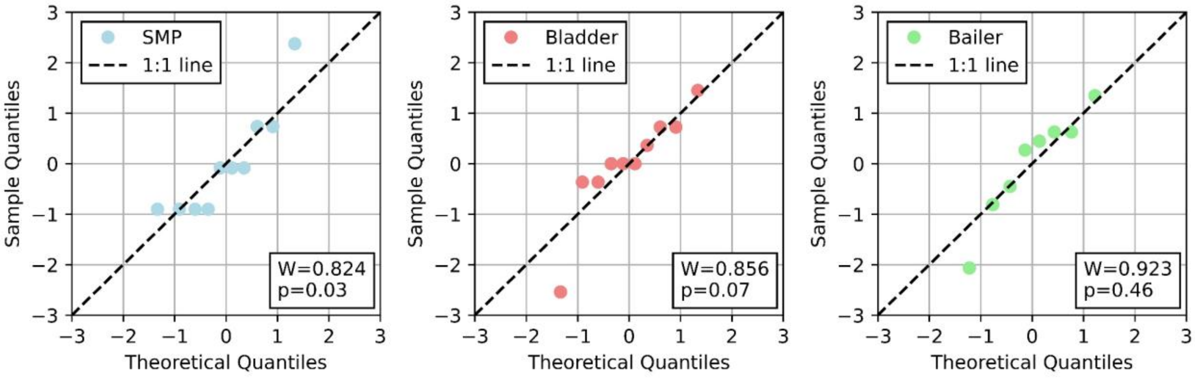

Figure S28. Q-Q plot of excess-N_2 concentrations for observation well 15. The test statistics (W) and the corresponding p-values of Shapiro-Wilk tests are provided.

Table S1. Major Ion Concentrations of the Samples collected at Observation Well 15. SMP – submersible pump.

Method	Na^+ mg/L	K^+ mg/L	Ca^{2+} mg/L	Mg^{2+} mg/L	Cl^- mg/L	NO_3^- mg/L	SO_4^{2-} mg/L	HCO_3^- mg/L
SMP	12.8	2.3	27.1	4.8	29.2	<0.3	69.2	11.9
Bladder	12.8	2.4	27.5	4.9	29.1	<0.3	70.7	13.3
Bailer	12.5	2.3	24.6	4.0	24.7	<0.3	53.7	24.9

Table S2. Descriptive statistics of major ion, N_2, Ar, and excess-N_2 concentrations for observation wells 1 to 14.

Variable	Group	N	Mean mg/L	Std. Dev. mg/L	Median mg/L	Min mg/L	Max mg/L
N_2	SMP	14	28.7	6.0	26.6	19.8	39.8
	Bladder	14	24.9	3.8	24.2	18.8	32.5
Ar	SMP	14	0.76	0.04	0.75	0.69	0.82
	Bladder	14	0.72	0.06	0.72	0.59	0.80
Excess-N_2	SMP	14	7.2	7.1	5.3	-0.2	21.1
	Bladder	14	5.0	5.4	3.8	-1	16.0
Na^+	SMP	14	15.4	7.6	18.3	5.0	23.5
	Bladder	14	15.3	7.5	18.0	5.1	23.1
K^+	SMP	14	3.7	2.5	3.0	0.8	9.4
	Bladder	14	3.6	2.5	2.9	0.8	9.6
Mg^{2+}	SMP	14	9.4	4.7	7.8	2.3	19.3
	Bladder	14	9.4	4.6	8.3	2.3	18.7
Ca^{2+}	SMP	14	55.6	17.2	57.7	19.5	82.4
	Bladder	14	56.6	17.5	58.2	20.8	86.8
Cl^-	SMP	14	36.0	21.9	44.0	5.5	61.4
	Bladder	14	35.4	21.1	44.1	6.1	57.2
NO_3^-	SMP	14	28.1	41.3	8.8	0.2	141.0
	Bladder	14	25.7	36.7	7.1	0.2	121.7
SO_4^{2-}	SMP	14	95.7	71.6	108.2	7.7	234.1
	Bladder	14	97.2	71.0	108.0	8.0	234.6
HCO_3^-	SMP	14	56.1	64.2	24.8	8.5	219.1
	Bladder	14	57.8	64.7	23.7	8.8	223.2

Table S3. Results of paired t-tests for major ion, N_2, Ar, and excess-N_2 concentrations for the observation wells 1 to 14.

Variable	t-Statistic	df	p-Value	Mean Difference mg/L		Cohens d
N_2	4.217	13	0.001	3.81	SMP > Bladder	1.13
Ar	4.092	13	0.001	0.04	SMP > Bladder	1.09
Excess-N_2	3.629	13	0.003	2.17	SMP > Bladder	0.97
Na^+	0.870	13	0.40	0.14	SMP > Bladder	-
K^+	0.538	13	0.60	0.04	SMP > Bladder	-
Mg^{2+}	0.626	13	0.54	-0.07	SMP < Bladder	-
Ca^{2+}	1.399	13	0.19	-1.08	SMP < Bladder	-
Cl^-	1.563	13	0.14	0.62	SMP > Bladder	-
NO_3^-	1.682	13	0.12	2.36	SMP > Bladder	-
SO_4^{2-}	1.455	13	0.17	-1.62	SMP < Bladder	-
HCO_3^-	1.099	13	0.29	-1.65	SMP < Bladder	-

Table S4. Results of paired Wilcox tests for major ion, N_2, Ar, and excess-N_2 concentrations for the observation wells 1 to 14.

Variable	V-Statistic	df	p-Value	Hodges-Lehmann Estimator mg/L		Effectsize r
N_2	5	13	0.001	3.80	SMP > Bladder	0.80
Ar	5	13	0.005	0.04	SMP > Bladder	0.79
Excess-N_2	12	13	0.012	2.25	SMP > Bladder	0.68
Na^+	27	13	0.37	0.09	SMP > Bladder	-
K^+	39	13	0.43	0.05	SMP > Bladder	-
Mg^{2+}	52.5	13	0.65	-0.09	SMP < Bladder	-
Ca^{2+}	69.5	13	0.30	-0.85	SMP < Bladder	-
Cl^-	26.5	13	0.20	0.40	SMP > Bladder	-
NO_3^-	2	13	0.05	3.15	SMP > Bladder	-
SO_4^{2-}	73	13	0.06	-0.96	SMP < Bladder	-
HCO_3^-	74	13	0.19	1.25	SMP < Bladder	-

Table S5. Descriptive statistics of major ion, N_2, Ar, and excess-N_2 concentrations for observation well 15.

Variable	Group	N	Mean mg/L	Std. Dev. mg/L	Median mg/L	Min mg/L	Max mg/L	CV %
N_2	SMP	10	31.8	0.14	31.8	31.5	32.0	0.4
	Bladder	10	29.1	0.46	29.2	28.0	29.5	1.6
	Bailer	8	29.4	0.79	29.6	27.7	30.4	2.7
Ar	SMP	10	0.73	0.004	0.73	0.72	0.73	0.6
	Bladder	10	0.69	0.01	0.69	0.68	0.70	0.8
	Bailer	8	0.72	0.01	0.72	0.71	0.73	1.0
Excess-N_2	SMP	10	11.7	0.13	11.7	11.6	12.0	1.1
	Bladder	10	10.6	0.29	10.6	9.9	11.0	2.7
	Bailer	8	9.5	0.60	9.65	8.3	10.2	6.3

Table S6. Results of Shapiro-Wilk tests for N_2, Ar, and excess-N_2 concentrations for observation well 15.

Variable	Group	W-Statistic	p-Value
N_2	SMP	0.825	0.03
	Bladder	0.838	0.04
	Bailer	0.891	0.24
Ar	SMP	0.509	0.000005
	Bladder	0.752	0.004
	Bailer	0.827	0.06
Excess-N_2	SMP	0.824	0.03
	Bladder	0.856	0.07
	Bailer	0.923	0.46

Table S7. Results of Levene's tests for N_2, Ar, and excess-N_2 concentrations for observation well 15.

Variable	df	F-Statistic	p-Value
N_2	2	2.478	0.10
Ar	2	0.893	0.42
Excess-N_2	2	3.502	0.04

Table S8. Results of Fligner-Killeen tests for N_2, Ar, and excess-N_2 concentrations for observation well 15.

Variable	df	Chi-Statistic	p-Value
N_2	2	6.131	0.05
Ar	2	2.484	0.29
Excess-N_2	2	5.361	0.07

Table S9. Results of one-factor Analyses of Variance for N_2, Ar, and excess-N_2 concentrations for observation well 15.

Variable	df	F-Statistic	p-Value
N_2	2	189.6	1e-9
Ar	2	150.1	1e-10
Excess-N_2	2	102.7	2e-8

Table S10. Results of one-factor Kruskal-Wallis tests for N_2, Ar, and excess-N_2 concentrations for observation well 15.

Variable	df	H-Statistic	p-Value
N_2	2	19.7	0.00005
Ar	2	21.5	0.00002
Excess-N_2	2	23.8	0.000007

Table S11. Results Tukey's HSD Multiple Comparison tests for N_2, Ar, and excess-N_2 concentrations for observation well 15.

Variable	Pair	q-Statistic	p-Value
N_2	SMP-Bladder	17.4	1e-11
	SMP-Bailer	14.7	5e-10
	Bladder-Bailer	1.7	0.46
Ar	SMP-Bladder	21.8	1e-13
	SMP-Bailer	2.9	0.12
	Bladder-Bailer	17.7	9e-12
Excess-N_2	SMP-Bladder	9.5	1e-6
	SMP-Bailer	18.3	4e-12
	Bladder-Bailer	9.3	2e-6

Table S12. Results Games-Howell Multiple Comparison tests for N_2, Ar, and excess-N_2 concentrations for observation well 15.

Variable	Pair	q-Statistic	p-Value
N_2	SMP-Bladder	18.4	6e-9
	SMP-Bailer	8.8	0.0001
	Bladder-Bailer	0.9	0.64
Ar	SMP-Bladder	17.4	1e-11
	SMP-Bailer	1.9	0.17
	Bladder-Bailer	10.9	1e-7
Excess-N_2	SMP-Bladder	11.0	2e-7
	SMP-Bailer	10.5	0.00002
	Bladder-Bailer	5.0	0.002

Table S13. Results Pairwise Wilcox Multiple Comparison tests for N_2, Ar, and excess-N_2 concentrations for observation well 15.

Variable	Pair	V-Statistic	p-Value	Hodges-Lehmann Estimator mg/L
N_2	SMP-Bladder	57	0.0005	2.70
	SMP-Bailer	0	0.0006	2.30
	Bladder-Bailer	0	0.14	-0.30
Ar	SMP-Bladder	0	0.0002	0.04
	SMP-Bailer	22	0.07	0.01
	Bladder-Bailer	80	0.0004	-0.03
Excess-N_2	SMP-Bladder	0	0.0005	1.10
	SMP-Bailer	0	0.0006	2.10
	Bladder-Bailer	1	0.0006	1.00

Table S14. Results Dunn's Multiple Comparison tests for N_2, Ar, and excess-N_2 concentrations for observation well 15.

Variable	Pair	Z-Statistic	p-Value
N_2	SMP-Bladder	4.3	0.00005
	SMP-Bailer	3.1	0.003
	Bladder-Bailer	1.0	0.32
Ar	SMP-Bladder	4.5	0.00002
	SMP-Bailer	1.1	0.28
	Bladder-Bailer	3.1	0.002
Excess-N_2	SMP-Bladder	2.8	0.01
	SMP-Bailer	4.8	4e-6
	Bladder-Bailer	2.3	0.02
